# Eccrine sweat gland carcinoma

**DOI:** 10.1016/S1808-8694(15)31081-8

**Published:** 2015-10-22

**Authors:** Fernando Danelon Leonhardt, Alessandra Zanoni, Fabiana Ponce, Leonardo Haddad, Cristovam Scapulatempo Neto, Onivaldo Cervantes, Márcio Abrahão

**Affiliations:** 1 M.S. - Department of Otorhinolaryngology - Head and Neck Surgery - UNIFESP-EPM. Otorhinolaryngologist - Head and Neck Surgeon. Mailing Address: Fernando Danelon Leonhardt - Rua Joaquim Floriano 72 cj. 47 Itaim-Bibi São Paulo SP 04534-000. Tel. (0xx11) 3079-3502 Fax: (0xx11) 3079-0731; 2 Fellow in Otology - Department of Otorhinolaryngology - Head and Neck Surgery - UNIFESP-EPM. Otorhinolaryngologist.; 3 MD. 3rd year Resident Physician - Department of Otorhinolaryngology - Head and Neck Surgery - UNIFESP-EPM.; 4 M.S. - Department of Otorhinolaryngology - Head and Neck Surgery - UNIFESP-EPM. Otorhinolaryngologist - Head and Neck Surgeon.; 5 Pathologist - Pathology Department - UNIFESP-EPM.; 6 Associate Professor - Department of Otorhinolaryngology - Head and Neck Surgery - UNIFESP-EPM. Head of the Head and Neck Surgery Discipline - Department of Otorhinolaryngology - Head and Neck Surgery - UNIFESP-EPM.; 7 Associate Professor - Department of Otorhinolaryngology - Head and Neck Surgery - UNIFESP-EPM. Associate head of the Head and Neck Surgery Discipline - Department of Otorhinolaryngology - Head and Neck Surgery - UNIFESP-EPM.; 8 Head and Neck Surgery Discipline - Department of Otorhinolaryngology - Head and Neck Surgery - UNIFESP-EPM.

**Keywords:** eccrine carcinoma, skin cancer, sweat gland

## INTRODUCTION

The differential diagnosis among the most frequent skin tumors, base-cell carcinoma and squamous-cell carcinoma, and the rare types, the sweat gland carcinomas, is fundamental for the early diagnosis and prognosis of a patient. These are classified as eccrine and apocrine, and the eccrine is the most common type. The eccrine sweat glands are most abundant in the palms of the hands and the feet soles, the forehead and the axilae.^1^

## CASE REPORT

R.S., 40 years of age, male, tan skin color, noticed a lump in his scalp, in the right temporal region, that had been there for about 30 years, having grown farther in one year, associated with an increase in cervical volume on the right side in the last 5 months. In the exam we noticed a lesion in his scalp, on the right temporal region, pinkish, sprouty, round and sessile and painless at palpation, measuring 7×5cm, not adhered to superficial or deep planes. A right side neck mass, in the posterior neck, fibro elastic, painful at palpation, measuring 2 × 1 cm. The temporal region biopsy and the neck tumor FNA showed a not-well differentiated adenocarcinoma, and the FNA suggested carcinoma metastasis. After tumor resection and right side modified radical neck clearance, and of right side neck posterior node chain clearance, a local flap was rotated in order to cover the wound. Pathology showed it to be an eccrine sweat gland carcinoma, with free margins and the presence of an eccrine sweat gland carcinoma in one of the 63 lymphnodes dissected (Figure 1). Because of this metastasis to a neck lymphnode, the patient was referred to radiotherapy; however the patient decided not to go through with it. The patient completed 2 years and 3 months of post-operative, without signs of local-regional or distant recurrence.

**Figure:**
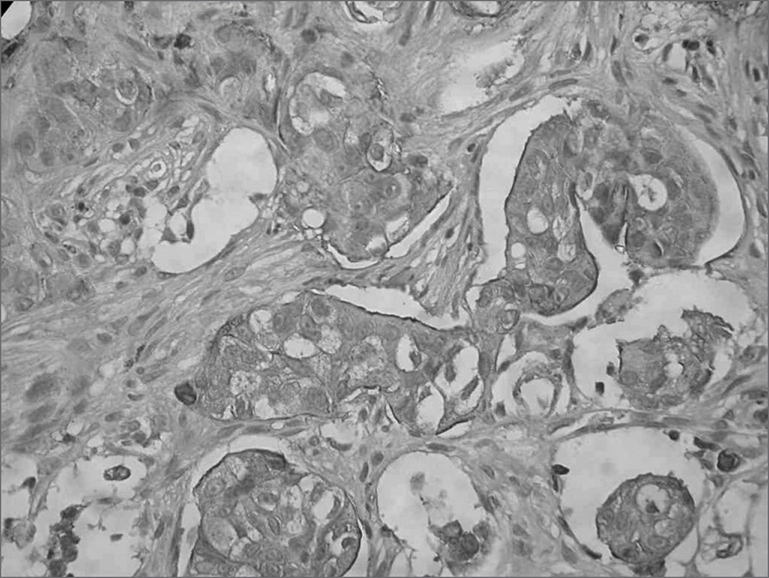
**Immunohistochemistry** - Immunohistochemical study showing a diffuse positiveness for CEA (Carcino-Embrionary Antigen), which is positive in eccrine annex tumors.

## DISCUSSION

Sweat gland carcinomas are rare neoplasias that affect men and women alike, predominating between 50 and 80 years of age. These tumors may be eccrine or apocrine and are very similar to other neoplasias, such as the skin adenocarcinoma and the basal cell carcinoma.^2^,^3^ The microscopic analysis may reveal cellular pleomorphism, tumoral cells networks and islets, irregular nucleus and abnormal chromatin pattern, high rates of mitosis and deep structure invasion, including nerves.4 It is a slow growth tumor for many years and that suddenly starts growing fast.^5^,^6^ Metastases are frequent and occur mainly to regional nodes, and also to the skin, bones and lungs.^5^ Malignant skin tumors may be treated by cryosurgery, curettage, surgery and surgery when tumor margins are frozen during surgery (Mohs procedure), with recurrence rates of 7 to 11% for the first three options and of 2 to 5% in the latter. Treatment of choice is broad surgical exeresis of the lesion, freezing the margins and radio and chemotherapy must be considered for patients with metastatic disease. The patient aforementioned underwent surgical exeresis of his temporal lesion and neck nodes clearance. As we confirmed the result of eccrine carcinoma with metastasis to the neck node, he was referred to radiotherapy, which was not carried out because for the patient's own desire. He has kept coming for follow up now for 2 years and 3 months, disease free.

## References

[1] Sampaio S.A.P., Rivitti E.A. (2001). Dermatologia..

[2] DeVita Jr VT, Hellman S, Rosenberg SA (2000). Cancer - Principles & Practice of Oncology..

[3] Arnold H, Odom R, James W (1994). Doenças da Pele de Andrews - Dermatologia Clínica. Em: Nevos, neoplasmas e cistos epidérmicos..

[5] Panet-Raymond G, Johnson WC (1973). Adenocarcinoma of the Eccrine Sweat Gland.. Arch Dermatol.

[6] Morris DM (1986). Carcinoma of Eccrine Sweat Gland: Experience with chemotherapy, autopsy findings in a patient with metastatic eccrine carcinoma, and a review of the literature.. J Surg Oncol.

